# Why are women not online? A study protocol to understand gender-based digital exclusion among marginalized women in Pakistan and promote digital gender inclusion

**DOI:** 10.1371/journal.pone.0356104

**Published:** 2026-08-20

**Authors:** Anam Shahil-Feroz, Muhammad Asim, Sana Liaquat, Salima Meherali, Saleema Allana, Bipasha Baruah

**Affiliations:** 1 Arthur Labatt Family School of Nursing, The University of Western Ontario, London, Ontario, Canada; 2 Department of Community Health Sciences, Aga Khan University, Karachi, Pakistan; 3 Faculty of Nursing, University of Alberta, Edmonton, Alberta, Canada; 4 Department of Gender, Sexuality and Women’s Studies, The University of Western Ontario, London, Ontario, Canada; Public Library of Science, UNITED KINGDOM OF GREAT BRITAIN AND NORTHERN IRELAND

## Abstract

**Background:**

Gender-based digital exclusion remains a major challenge in Pakistan, where women are significantly less likely than men to own mobile phones or access mobile internet. Barriers include family disapproval, sociocultural and religious restrictions, safety concerns, and patriarchal norms that limit women’s autonomy. Although government initiatives have expanded digital access and literacy, they largely focus on individual-level solutions and do not adequately address the social and structural factors shaping women’s digital exclusion.

**Goal and Objectives:**

This study aims to explore the sociocultural norms and practices influencing marginalized women’s access to and use of digital technologies in a low-income community on the outskirts of Karachi, Pakistan. Specifically, the study seeks to: (1) identify barriers and facilitators influencing women’s access to and use of digital technologies; (2) explore how women, family members, and community leaders perceive and influence women’s access to and use of digital technologies; and (3) co-design community-informed strategies to promote digital gender inclusion.

**Methods and Analysis:**

Guided by Technofeminism and community-based participatory research (CBPR) principles, this qualitative study will be conducted in Azam Basti, Karachi. The study comprises three phases: (1) establishment of a Community Advisory Committee to guide the research; (2) focus group discussions and in-depth individual interviews with marginalized women, family gatekeepers, and community leaders using photo-elicitation and role-playing methods to explore gendered norms and technology access; and (3) co-design workshops to develop community-informed solutions for reducing the digital gender gap. Data will be analysed using reflexive thematic analysis.

**Discussion:**

This study will provide insights into how sociocultural norms and patriarchal structures shape women’s digital exclusion in Pakistan. By engaging family gatekeepers and community leaders, it moves beyond awareness-based approaches to co-develop sustainable strategies for digital inclusion. The research methods, process, and findings from this study will be valuable to researchers and policymakers seeking to address the digital gender gap by providing evidence to inform the development of contextually appropriate strategies for promoting digital inclusion among marginalized women.

## Introduction

The United Nations (UN) defines digital inclusion as “equitable, meaningful, and safe access to use, lead, and design of digital technologies, services, and associated opportunities for everyone, everywhere” [1]. However, achieving digital inclusion remains challenging due to persistent gender-based digital exclusion, which refers to disparities in access to and use of Information and Communication Technologies (ICTs) between men and women [2,3]. Despite increasing digitalization initiatives, women in low- and middle-income countries (LMICs) continue to lag behind in mobile phone ownership and usage [4]. While it is often assumed that this gap will close naturally as digital technologies become more widespread, the reality is that men and women engage with technology differently due to gendered social norms, which change far more slowly than technology itself [5]. As a result, women continue to face barriers that limit their ability to benefit from digital opportunities [3,6,7]. Addressing these barriers and advancing digital gender inclusion can yield significant benefits, including enhanced agency [8], connectivity, safety, quality of life [9] and improved access to essential resources like health information, financial services, and employment opportunities [6,10,11].

These challenges are particularly pronounced in LMICs such as Pakistan, where concerns about the digital gender divide remain significant [6]. Pakistan, the fifth most populous country with over 220 million people and 49 percent of the women population, ranks 145^th^ out of 146 countries in the Global Gender Gap Index 2024 [12]. This represents a decline from its 142^nd^ position in 2023, highlighting growing challenges in achieving gender parity [12]. Despite rapid growth in mobile technology, women in Pakistan continue to face substantial inequalities in mobile ownership and internet access [9]. According to the Global Systems for Mobile Communications Association’s (GSMA) Mobile Gender Gap report 2024, women in Pakistan are 38% less likely than men to own a mobile phone or access mobile internet [13]. Surveys and consultations conducted by the Pakistan Telecommunication Authority (PTA) as part of its ‘*digital inclusion strategy’* [4] development process revealed that 16% of female respondents and 23% of male respondents believed women should not use mobile phones at all [4]. The surveys also found that some women accessed phones by sharing or borrowing devices, with many men perceiving that women could not use them safely [4]. Women in rural areas and those from low socioeconomic backgrounds remain the most disadvantaged in terms of mobile phone ownership and usage, as reported in the Pakistan Social and Living Standards Measurement (PSLM) survey conducted by the Pakistan Bureau of Statistics (PBS) [14,15].

This digital gender divide stems from barriers related to access, affordability, education, technological literacy, safety and security concerns, as well as demographic, cultural, and religious restrictions [6,8,9,16]. Among these, ‘*deeply entrenched sociocultural barriers’* are the most significant yet critically underexplored factor limiting marginalized women’s access to and use of digital technologies [6,9,17]. In this context, marginalized women are women who experience intersecting social, economic, and gender-based disadvantages including poverty, restrictive gender norms, limited decision-making power, constrained mobility, and restricted access to and control over digital technologies that limit their opportunities for digital participation. Global initiatives, including the EQUALS global partnership [18], have brought together more than 100 partners to bridge the digital gender divide. However, many of these efforts are led by women for women. Recognizing this gap, TEQtogether [19] was conceived to engage men in changing their behaviors and to address the structural constraints limiting women’s and girls’ participation in the digital technology sector. While most research in this area has focused on North American and European contexts [19], TEQtogether conducted a study in Pakistan in 2020 with university students and faculty in STEM (Science, Technology, Engineering, and Mathematics) fields to examine men’s attitudes and behaviors regarding women’s access to digital technologies [17]. The research highlights that sociocultural norms, traditional values, religion, and family customs limit women’s access to technology [17]. Male family members control technology access, fearing exposure to content that contradicts cultural beliefs and brings shame. Girls using the internet are often ostracized and considered unmarriageable. While men typically dominate decisions, elderly women also influence household dynamics and young women's technology access, reinforcing the need for attitudinal change from both genders [17].

The Government of Pakistan (GoP) has taken several steps to bridge the gender gap in mobile phone ownership and internet use through initiatives such as the ‘Digital Pakistan’, which promotes digital literacy, innovation, and entrepreneurship; the ‘Kamyab Jawan’ [20], which provides loans and training to young women entrepreneurs; and the ‘Telecom Policy 2015’, which aims to increase broadband penetration and promote affordable internet access for all [6]. However, these efforts primarily focus on improving digital access, literacy, and entrepreneurship [16,21,22]; yet they target urban areas and focus on individual-level solutions that, while necessary, remain insufficient. Additional initiatives must address systemic issues, including deeply entrenched patriarchal structures that significantly hinder marginalized women’s access to and use of digital technologies. Although family gatekeepers are increasingly recognized as allies in addressing these barriers, their involvement has largely been limited to public awareness campaigns. We argue that achieving digital gender inclusion requires a shift from awareness campaigns to active engagement with family gatekeepers and community leaders in co-designing sustainable solutions to positively influence the norms around technology access and usage.

Gender digital equity has emerged as a significant global concern, highlighted by substantial empirical and theoretical research [2,23–26] and initiatives led by national and international organizations, including GSMA, the International Telecommunication Union (ITU), PTA, and UN Women. LMICs have already lost over $1 trillion in GDP due to women's exclusion from the digital world [27], with women's digital inclusion in Pakistan being notably lower compared to regional peers [6,9,13]. Bridging the digital gender divide is essential to realizing women’s rights and achieving Sustainable Development Goals (SDGs), particularly #3 (good health and well-being), #5 (gender equality), and #10 (reduced inequality) [28]. Research consistently demonstrates strong positive links between women’s access to and use of digital technologies with economic growth, better health outcomes, women’s empowerment, increased labor force participation, and increased awareness of rights [2,4,15,23,25,26]. According to GSMA, closing the gender gap in mobile access and usage in LMICs could yield substantial economic growth, with an estimated $700 billion in GDP growth over five years [13].

### Aim

This project aims to explore the sociocultural norms and practices shaping women’s access to and use of digital technologies and engage marginalized women, family gatekeepers (such as male partners, brothers, male relatives, and elderly women), and community leaders in co-designing tailored solutions to promote digital gender inclusion. The findings from this project will make a meaningful contribution to progress towards SDG 5 on gender equality [29] and lay the foundation for digital inclusion strategies to help the Pakistani Government, Canadian agencies, and research organizations to bridge the gender digital divide.

### Research questions

The following research questions are central to this community-based participatory research (CBPR) project:

1. What barriers and facilitators influence women's access to and use of digital technologies in Azam Basti?
2. How do marginalized women, family gatekeepers, and community leaders perceive and shape women's access to and use of digital technologies?
3. How can interventions be co-designed with marginalized women, family gatekeepers, and community leaders to promote digital gender inclusion for marginalized women?

### Theoretical framework

This study is grounded in Judy Wajcman’s Technofeminism theoretical framework, which critically examines the intersection of gender and technology [30]. Wajcman argues that technology is not neutral; it is shaped by the social, cultural, and political contexts [31]. Building on feminist theory and Science and Technology Studies (STS), Technofeminism explores how gender and technology mutually shape each other—technology is not just a tool, but a reflection of and reinforcement to existing social structures and gender dynamics [32]. A central tenet of this framework is that both gender and technology influence one another, and that technologies are socially constructed, embedded within systems of power [33]. This perspective is particularly relevant for this study, which examines the digital gender gap among marginalized women in Pakistan, where sociocultural norms and patriarchal ideologies create significant barriers to women’s access to and use of digital technologies. These sociotechnical barriers go beyond mere access—they encompass the cultural narratives and power dynamics that shape how technology is perceived and used [31]. Furthermore, the framework’s emphasis on collaboration and inclusivity [32] aligns with the CBPR approach, which involves engaging not only marginalized women but also family gatekeepers.

## Materials and methods

### Study design

We will conduct a CBPR to explore the sociocultural norms and practices that influence women’s access to and use of digital technologies, and to engage marginalized women, community leaders, and family gatekeepers in co-designing tailored solutions to promote digital gender inclusion. CBPR is a research design that actively engages participants in addressing issues of concern to their community and promotes their participation in all phases of the research process [34–37]. It is anticipated that conducting CBPR with marginalized women, community leaders, and family gatekeepers as equal collaborators will enhance agency, empowerment, and greater uptake of research findings by the community. Our prior experience with high-risk pregnant women and family caregivers in designing a telemonitoring program demonstrated that CBPR ensures the development of user-centered solutions while fostering active involvement and ownership among participants [38,39]. Aga Khan University, our grant collaborator, has established a community-based Urban Health Program to provide health-education and referrals in Azam Basti since 2022. Their ongoing work emphasizes that improving women’s access to resources and opportunities in Azam Basti requires addressing patriarchal ideologies and structural barriers (recent consultation, Jan 2024).

### Research site

The project will be conducted at Azam Basti (Site Lead: MA). A significant proportion of residents belong to low-income households, with women mainly working as housewives, home-based domestic workers, beauticians, and local school teachers. The site offers a unique opportunity to explore the intersection of technology, gender, and power structures, as most women are illiterate, marginalized, less likely to be involved in decision-making, and face restrictive social norms, along with limited access to technology, education, healthcare, social services, and employment opportunities. The research will also be relevant to other groups, such as women in other LMICs and immigrant women in high-income countries who face digital gender exclusion.

### Study sample and recruitment

We will purposively sample [40,41] three participant groups for focus group discussions (FGDs), in-depth interviews, and co-design work, including marginalized women, family gatekeepers (such as male partners, brothers, male relatives, and elderly women), and community leaders. These groups play a crucial role in shaping access to and use of technology among marginalized women. The final number of in-depth interviews and FGDs will depend on theoretical saturation [42]. FGDs, in-depth interviews, and co-design work will take place at the field office of Urban Health Programme which is accessible to the PA [ASF] and Co-applicant [MA] through Aga Khan University’s ongoing community health program. A community liaison from Azam Basti will be hired to recruit and retain participants to ensure connection with trusted community members in research activities. In addition, we will recruit a senior researcher (female) and two research assistants (one male, one female) with relevant fieldwork experience in CBPR to support community engagement, participant recruitment, data collection (FGDs, in-depth interviews, and co-design work), and transcription.

### Phase 1 – Community advisory committee (CAC) formation

Prior to data collection and co-design work, the PA (ASF), co-applicant (MA), and a research team member (SL) will form an advisory committee consisting of two leaders from community, two marginalized women, and three family gatekeepers (two male partners and one elderly woman) from Azam Basti, nominated the by community liaison. This committee will co-lead the project alongside the research team and meet quarterly to provide input on research activities. The CAC will play a vital role in supporting FGDs, in-depth interviews, and co-design activities, such as co-developing the FGD and in-depth interview guides, selecting appropriate images for photo elicitation, planning role-play activities, brainstorming questions, and reviewing and validating analysis. Their involvement will ensure cultural relevance, accuracy, and alignment with community norms and values. There will be three sessions with CAC, and each session will last 90–120 minutes. The participants will receive a total of PKR 4500 (16 USD) for the compensation of their time.

### Phase 2 – Focus group discussions and in-depth interviews

This phase will involve FGDs and in-depth individual interviews with marginalized women, family gatekeepers, and community leaders to explore the barriers and facilitators influencing women's access to and meaningful use of digital technologies. The total sample across all participant groups will comprise approximately 36–45 participants (Table 1).

**Table 1:** Data collection methods, participants, and sample size.

Data Collection Method	Participants	Sample Size
FGDs	Marginalized women	2 FGDs (4–5 participants per group)
	Family gatekeepers	2 FGDs (4–5 participants per group)
	Community leaders	2 FGDs (4–5 participants per group)
In-depth interviews	Marginalized women	4–5 participants
	Family gatekeepers	4–5 participants
	Community leaders	4–5 participants

For the FGDs, two semi-structured discussion guides have been developed collaboratively by the research team and the CAC: a photo-elicitation guide for marginalized women and a role-play guide for family gatekeepers and community leaders. Two FGDs will be conducted with each participant group, with 4–5 participants per group (Table 1). Each discussion will last approximately 60–90 minutes and will be conducted in Urdu at the Urban Health Programme community health centre. Participants will be provided with complimentary refreshments. Recruitment will be coordinated by the community liaison and research team, and participatory facilitation techniques appropriate to each participant group will be used to encourage discussion. The community liaison and research staff will coordinate participant recruitment and use participatory facilitation techniques appropriate for each participant group.

*FGDs with marginalized women* will use photo-elicitation [43] to explore their lived experiences with technology access and sociocultural barriers. The guide comprises of 5–6 culturally accepted curated photos by the research team. These photos visually depict various aspects of technology access and sociocultural norms, such as digital devices (e.g., basic phones, smartphones), mobility restrictions (e.g., women unable to visit technology shops), and cultural expectations (e.g., women engaged in household chores or caregiving activities). These photos will serve as anchors for discussions, evoking emotions and eliciting deeper insights into how these factors shape women's access to and use of technology in their daily lives. For each image the facilitator follows a fixed three-step “ladder” of questioning that moves from the concrete to the personal to the analytic: participants are first asked to describe what they see; then to relate the image to their own lives; and finally, to interpret the underlying norms, decisions, or power relations it evokes. This sequence is adapted from the SHOWeD questioning protocol developed for photo voice [44]. The complete image set with its accompanying prompts is provided in S1 Appendix. All images are generated through Open AI ChatGPT image generation tool keeping in view of local cultural context. The images are non-identifiable, depicting no real community members, homes, or events and were reviewed by the CAC to use for data collection.

*FGDs with community leaders and family gatekeepers*—including leaders from local and development organizations, male partners, brothers, other male relatives, and older women— will use the same role-play guide [45] as a participatory method to explore gendered power dynamics, family values, and concerns about safety that shape decisions about women’s digital inclusion. In role-playing exercises, participants will act out different scenarios where decisions are made about women’s access to and use of technology. These role-playing activities will help uncover current practices, norms, and power structures within the community that influence community leaders’ and gatekeepers’ attitudes toward women’s digital participation. The development of these role-playing activities have been guided and supported by the CAC to ensure cultural relevance and accuracy. The guide includes three core scenarios reflecting common household and community situations related to women’s access to and use of digital technologies: (1) family decision-making regarding a woman’s access to a mobile phone, (2) community discussions about establishing a women-only digital skills programme, and (3) a household emergency in which a woman requires access to a mobile phone to seek assistance. Additional scenarios may be used flexibly depending on the participant group and discussion flow. Each scenario includes a brief, non-identifying case description, role cards, facilitation guidance, debriefing questions, and scenario-specific ethical considerations. Participants will enact each scenario for approximately 20 minutes, followed by a structured debrief to connect the role-play to real-life experiences and facilitate critical reflection on social norms, gender dynamics, and decision-making processes influencing women’s digital inclusion. The facilitator will observe the enactment and provide minimal prompts only when needed to support discussion. The complete role-play guide and debriefing scripts are provided in S2 Appendix.

In addition to the FGDs, 4–5 individual in-depth interviews will be conducted with participants from each of the three groups to explore sensitive and personal dimensions of digital exclusion in greater depth and within a private setting. In-depth interview participants will be purposively sampled through the community liaison using the same recruitment procedures as the FGDs. Each interview will last approximately 40–60 minutes and will be conducted in Urdu (or Sindhi, where required) at the Health Centre. Participants will receive refreshments in appreciation of their time. The interviews will follow semi-structured guides developed separately for marginalized women (S3 Appendix) and for family gatekeepers and community leaders (S4 Appendix).

### Analysis

The FGDs and in-depth interviews will be transcribed in their original language (Urdu) and then translated into English by bilingual researchers. The transcripts and visual data from photo-elicitation and role-playing will be co-analysed with the advisory committee, a method successfully used in our previous CBPR studies by the team [46–49]. The use of technofeminism framework [31,32] will allow us to explore how patriarchal structures and gendered social norms shape digital inclusion of marginalized women in Azam Basti. Braun and Clarke’s reflexive thematic analysis *[*50–52*]* approach will be employed, with two researchers independently coding the data after familiarizing themselves with the transcripts and visual materials. Codes will be organized and analysed using NVivo software [53]. Comparative analysis across participant groups will be conducted to examine similarities and differences in perceptions, power dynamics, and influences shaping women’s technology use. Throughout the process, the research team will engage in ongoing reflection to ensure the themes accurately reflect the data. The advisory committee will review and validate the themes to ensure they align with community perspectives. The findings from Phase 2 will inform co-design activities in Phase 3 to address gendered barriers to digital inclusion. The research team will maintain reflexive memos and audit trails to ensure transparency and rigor in the analysis process [54,55].

### Phase 3 – Co-design workshops

Phase 3 will involve co-design workshops with participants identified in Phase 2 to co-create strategies to positively influence the norms around technology access and usage. Additional participants will be recruited as needed through the same recruitment method. “Co-design is about engaging consumers and users of products and services in the design process…[to] ultimately lead to improvements and innovation” [56]. All participant groups including marginalized women, family gatekeepers, and community leaders will participate in 3 co-design workshops, depending on the complexity of the solutions and the level of engagement from participants [57]. Each workshop will consist of 8–10 participants, with a total of 25–30 participants across all groups. Each workshop will last approximately 90–120 minutes and will be conducted in Urdu at the field office of Urban Health Program of Aga Khan University, Karachi*.* Participants will be provided complimentary food and refreshment for their time.

Before conducting the co-design workshops, facilitators will establish clear ground rules to support respectful and equitable participation. To ensure that marginalized women are meaningfully included and able to contribute on an equal footing with family gatekeepers and community leaders, facilitators will set participation norms that explicitly encourage women to speak, protect their space to contribute, and have balanced engagement across all participants.

*Co-design workshops with marginalized women, family gatekeepers, and community leader*s will begin by revisiting the Phase 2 findings and identifying sociocultural barriers that limit women’s digital access. Participants will then engage in a brainstorming session [58] to co-develop 2–3 strategies aimed at enabling women’s access to mobile technology. For instance, the group may explore the possibility of developing new community norms that support women’s digital access. Followed by a traditional-style dialogue session inspired by the World Café method [59,60] to further develop and refine these strategies. Discussions prompted by questions such as: What benefits could mobile technology offer to women in our community? How might a male family member, like a brother or partner, support a woman in using mobile technology? What shifts in our perceptions of technology and women are necessary for mobile technology to be more widely accepted in our homes. These exercises will provide participants with an opportunity to experiment with more inclusive family dynamics in a low-risk environment.

A semi-structured co-design guide (S5 Appendix) will be developed by the research team in collaboration with the CAC to guide the brainstorming and World Café activities. The guide will include prompts to facilitate reflection on Phase 2 findings, generate strategies to address identified barriers, and support dialogue to refine and contextualize proposed solutions. Facilitator instructions and discussion prompts will be included to ensure meaningful participation and capture diverse perspectives throughout the co-design process. A key aspect of these workshops will be allowing participants to reflect on their feelings regarding the co-designed solutions and the potential challenges or benefits they foresee. The goal of these workshops is to co-create solutions that not only enable marginalized women’s digital inclusion but also reshape the underlying power dynamics that have historically restricted it.

### Analysis

The findings from each workshop will be transcribed in their original language (Urdu/) and then translated into English for reflexive thematic analysis [50–52]. In the first round, two researchers will independently code the workshop data to identify patterns in the co-designed solutions. Later, the team and CAC will review the data and refine these solutions to develop digital inclusion strategies to support the Pakistani government, Canadian agencies, and research organizations in bridging gender digital divide for marginalized women in Azam Basti and Pakistan.

### Study timeline

At the time of manuscript submission, the study has not yet commenced, and no participant recruitment or data collection has taken place. The two-year project is scheduled to begin in February 2026. During the first six months (February–July 2026), project preparation activities will include establishment of the CAC, community engagement, development and pilot testing of study materials, ethics approvals, and recruitment and training of research staff. Participant recruitment is expected to begin in August 2026 and be completed by November 2026. Data collection, including FGDs and co-design workshops, is expected to be completed by April 2027. The remaining project period (May 2027–January 2028) will focus on data analysis, manuscript preparation, policy brief development, knowledge mobilization, community dissemination activities, and development of culturally appropriate resources. Study results are expected to be available beginning in late 2027, following completion of the planned analyses. The CAC will meet throughout the project to provide ongoing guidance and ensure community relevance and engagement.

### Ethical considerations

Ethical approval for this project has been obtained from the Western University Non-Medical Research Ethics Board (NMREB) (Protocol # 127689), Aga Khan University (Protocol # 2026-12467-40149), and the National Bioethics Committee in Pakistan (Protocol #4–87/NBCR-1413/25–26/1938).

Written informed consent will be obtained from all participants, including CAC members, marginalized women, family gatekeepers, and community leaders, prior to participation in any study activities. Consent procedures will be conducted in Urdu, with study information provided in plain language to ensure comprehension. Participants will be informed about the study purpose, procedures, expected duration, number of sessions, voluntary nature of participation, their right to decline any question, take breaks, or withdraw at any time without consequence. Information regarding compensation, potential risks and benefits, confidentiality protections and their limitations in group settings, data storage and retention procedures, and investigator contact details will also be provided. Consent will be documented through a signed written consent form. For participants who are unable to read or write, the consent information will be read aloud verbatim in Urdu, and consent will be documented using a thumbprint, witnessed by an impartial individual independent of the research team. Participants will have the opportunity to ask questions and sufficient time to consider participation before providing consent.

## Discussion

This study will advance understanding of gender-based digital exclusion by examining how sociocultural norms and patriarchal ideologies shape marginalized women’s access to and use of digital technologies. By applying a participatory approach, this research will generate methodological contributions through the engagement of family gatekeepers and community leaders – actors who are often overlooked in digital inclusion initiatives but play a critical role in influencing women’s technology access and use. Furthermore, this study will extend the application of Technofeminism within LMIC contexts, particularly Pakistan, by exploring how gender, power relations, and social structures influence digital participation among marginalized communities.

A key contribution of this study is the intentional involvement of family gatekeepers, including male partners, brothers, and elderly women, in discussions surrounding gender-based digital exclusion. Recognizing that household-level decision-making and social norms significantly influence women’s access to technology, this approach moves beyond individual-level interventions focused solely on women’s digital literacy and access. Through community engagement activities, arts-based knowledge translation materials (including videos, infographics, and voice messages), and stakeholder dialogues, the study will support greater awareness and understanding of the social factors shaping women’s digital inclusion in Azam Basti and similar settings.

The findings will provide evidence to inform the development of contextually appropriate strategies to promote digital gender inclusion among marginalized women. The stakeholder report and policy brief developed through this project will offer actionable recommendations for organizations such as the Pakistan Telecommunication Authority, the Ministry of Information Technology, and relevant national and international partners, supporting broader efforts to address gender disparities in digital access and use. By centering community perspectives and co-designing solutions with key stakeholders, this research has the potential to inform more sustainable and socially responsive digital inclusion initiatives.

Finally, this study will contribute to efforts aimed at advancing women’s rights and achieving the SDGs, particularly SDG #3 (good health and well-being), SDG #5 (gender equality), and SDG #10 (reduced inequality). The findings will also support the work of collaborators such as Aga Khan University in strengthening women’s empowerment initiatives in underserved communities surrounding Karachi and provide insights for organizations working to advance digital gender inclusion across Pakistan. Additionally, the project will provide graduate students with valuable experience in participatory research with marginalized communities, strengthening their research capacity and professional development.

### Limitations

This study has several limitations that should be considered when interpreting its findings. First, as the research will be conducted within a single marginalized community in Azam Basti, Karachi, the findings may not be directly generalizable to all women and communities across Pakistan or other LMIC contexts. However, the participatory approach and in-depth exploration of community experiences will provide valuable contextual insights that may inform similar settings. Second, discussions of gender norms, technology use, and household decision-making may be influenced by social desirability bias, as participants may underreport or modify their perspectives due to cultural sensitivities surrounding gender roles and technology access. Efforts to mitigate this limitation will include creating safe discussion environments, engaging diverse participants, and using community-informed approaches. Third, while involving family gatekeepers and community leaders represents an important step toward addressing structural barriers, changing deeply embedded sociocultural norms requires sustained engagement beyond the duration of the project. Future research should examine the long-term effectiveness and scalability of co-designed interventions developed through this study.

## Conclusion

Gender-based digital exclusion remains a significant barrier to women’s empowerment, particularly in LMICs where sociocultural norms and power structures continue to shape access to and use of digital technologies. This study addresses an important gap by moving beyond individual-level approaches and engaging family gatekeepers and community leaders as key actors in shaping more inclusive digital environments. Through participatory methods and co-design approaches, this research will generate contextually relevant insights into the social and structural factors influencing marginalized women’s digital participation in Azam Basti, Pakistan. The findings will contribute to theoretical and methodological advancements in understanding the gender digital divide and provide evidence to inform policies, programs, and community-based interventions aimed at advancing digital gender inclusion. By recognizing the role of households and communities in shaping women’s technology access, this study represents an important step toward creating more equitable digital opportunities for marginalized women.

## Supporting information

S1 Appendix Photo-elicitation focus group discussion guide for marginalized women. (DOCX)

S2 Appendix Role-play focus group discussion scenarios for family gatekeepers and community leaders. (DOCX)

S3 Appendix In-depth interview guide for marginalized women. (DOCX)

S4 Appendix In-depth interview guide for family gatekeepers and community leaders. (DOCX)

S5 Appendix Co-design workshop guide. (DOCX)

## References

[1] Nations U. Roundtable on Digital Inclusion. https://www.un.org/techenvoy/sites/www.un.org.techenvoy/files/general/Definition_Digital-Inclusion.pdf

[2] Singh S. Bridging the gender digital divide in developing countries. Journal of Children and Media. 2017;11(2):245–7. doi: 10.1080/17482798.2017.1305604

[3] Foundation IS. What’s the Gender Digital Divide? 2024. https://www.isocfoundation.org/2024/10/whats-the-gender-digital-divide/

[4] Pakistan Telecommunication Authority U. Digital gender inclusion strategy. 2024. https://www.pta.gov.pk/assets/media/digital_gender_inclusion_strategy_28-02-2024.pdf

[5] Highet C, Salman A, Singh N. The digital gender divide won’t close by itself–here’s why. Washington, DC: Consultative Group to Assist the Poor (CGAP). 2020.

[6] Akram M. Digitalisation and Women in Pakistan. Pakistan: National Commission on the Status of Women and United Nations Development Programme. 2023.

[7] Shefa A. The use of mobile technology and mobile applications as the next paradigm in development: can it be a game-changer in development for women in rural Afghanistan?. University of Southern California. 2014.

[8] Khurshid A. Gender equality and inclusive technology: cultural innovations for sustainable development in Khyber Pakhtunkhwa (KP), Pakistan. Pakistan Journal of Gender Studies. 2024;24(2):107–23.

[9] Jamil S. From digital divide to digital inclusion: Challenges for wide-ranging digitalization in Pakistan. Telecommunications Policy. 2021;45(8):102206. doi: 10.1016/j.telpol.2021.102206

[10] GSMA. Policy considerations to accelerate digital inclusion for women in low- and middle-income countries. 2022. https://www.gsma.com/solutions-and-impact/connectivity-for-good/mobile-for-development/wp-content/uploads/2022/09/Policy-considerations-to-accelerate-digital-inclusion-for-women-in-low-and-middle-income-countries-report.pdf

[11] Youngs G. Cinderella or cyberella? empowering women in the knowledge society. Taylor & Francis. 2007.

[12] Forum WE. Global Gender Gap 2024. 2024. https://www3.weforum.org/docs/WEF_GGGR_2024.pdf

[13] Association’s GSfMC. The Mobile Gender Gap Report 2024. 2024. https://www.gsma.com/r/wp-content/uploads/2024/05/The-Mobile-Gender-Gap-Report-2024.pdf?utm_source=website&utm_medium=button&utm_campaign=gender-gap-2024

[14] Women U. National report on the status of women in Pakistan. 2023. https://pakistan.unwomen.org/sites/default/files/2024-06/pk-c972-national-report-on-the-status-of-women-s.pdf

[15] Amber H, Chichaibelu BB. Narrowing the gender digital divide in Pakistan: Mobile phone ownership and female labor force participation. Review Development Economics. 2023;27(3):1354–82. doi: 10.1111/rode.12994

[16] Aslam A, Abidi SNM, Rizvi SSA. Digital Literacy and Women’s Empowerment: Bridging the Gender Gap in Technology for Achieving Sustainable Development Goals (SDGs). Pakistan Journal of Gender Studies. 2024;24(2):93–106.

[17] Unwin T, Gardezi A. Male attitudes and behaviours towards women and digital technologies in Pakistan.

[18] Organization DC. EQUALS Global Partnership. 2004. https://www.equalsintech.org/

[19] Unwin TQ, Liz TEQ. 2024. https://teqtogether.org/

[20] Shahid U. Study on the design of business incubation centres: for the government of Pakistan’s Kamyab Jawan Programme. 2021.

[21] Iqbal I, Atay T, Savitskaya A. Digital literacy gender gap in e-education through social media during the COVID-19 lockdown in Pakistan and Turkey. Research Anthology on Remote Teaching and Learning and the Future of Online Education. IGI Global. 2023. 2103–24.

[22] Irfan T, Salam MT. Kaarvan Crafts Foundation: Embracing Digital Literacy for Women Empowerment. Emerald Emerging Markets Case Studies. 2020;10(4):1–34.

[23] Co-operation O f. E. Bridging the digital gender divide: Include, upskill, innovate. OECD. 2018.

[24] Pawluczuk A, Lee J, Gamundani AM. Bridging the gender digital divide: an analysis of existing guidance for gender digital inclusion programmes’ evaluations. Digital Policy, Regulation and Governance. 2021;23(3):287–99.

[25] Liff S. An evolving gender digital divide?. OII Internet Issue Brief. 2004.

[26] Antonio A, Tuffley D. The Gender Digital Divide in Developing Countries. Future Internet. 2014;6(4):673–87. doi: 10.3390/fi6040673

[27] Padmavathy N, Kandavel R. Sustainable inclusion of women in digital India. Atlantis Press. 2024.

[28] Kerras H, Sánchez-Navarro JL, López-Becerra EI, de-Miguel Gómez MD. The Impact of the Gender Digital Divide on Sustainable Development: Comparative Analysis between the European Union and the Maghreb. Sustainability. 2020;12(8):3347. doi: 10.3390/su12083347

[29] Hirsu L, Hashemi L, Quezada-Reyes Z. SDG 5: Achieve gender equality and empower all women and girls. RMIT University. 2019. https://www.rmit.edu.au/content/dam/rmit/rmit-images/college-of-dsc-images/eu-centre/sdg-5-policy-brief.pdf

[30] Wajcman J. Gender and work: a technofeminist analysis. Handbook of gender, work and organization. 2011. 263–75.

[31] Wajcman J. Feminist theories of technology. Cambridge Journal of Economics. 2010;34(1):143–52.

[32] Gill R. Technofeminism. Science as Culture. 2005;14(1):97–101.

[33] Wajcman J. Reflections on gender and technology studies: In what state is the art?. Social Studies of Science. 2000;30(3):447–64.

[34] Minkler M, Wallerstein N. Community-based participatory research for health: from process to outcomes. John Wiley & Sons. 2011.

[35] Reason P. Handbook of action research: Participative inquiry and practice. Sage. 2001.

[36] Kemmis S, McTaggart R, Nixon R. The action research planner: Doing critical participatory action research. Springer. 2014.

[37] McTaggart R. Principles for participatory action research. Adult Education Quarterly. 1991;41(3):168–87.

[38] Shahil-Feroz A, Riaz A, Yasmin H, Saleem S, Bhutta Z, Seto E. Perceived barriers and facilitators of implementing a sustained smartphone-based telemonitoring program for pregnant women at high-risk for pre-eclampsia in the public and private sectors in Pakistan. Digit Health. 2024;10:20552076241292682. doi: 10.1177/20552076241292682 39659397 PMC11629423

[39] Feroz AS, De Vera K, D Bragagnolo N, Saleem S, Bhutta Z, Seto E. Understanding the Needs of a Mobile Phone-Based Telemonitoring Program for Pregnant Women at High Risk for Pre-Eclampsia: Interpretive Qualitative Description Study. JMIR Form Res. 2022;6(2):e32428. doi: 10.2196/32428 35200152 PMC8914731

[40] Ahmad M, Wilkins S. Purposive sampling in qualitative research: a framework for the entire journey. Qual Quant. 2024;59(2):1461–79. doi: 10.1007/s11135-024-02022-5

[41] Nyimbili F, Nyimbili L. Types of Purposive Sampling Techniques with Their Examples and Application in Qualitative Research Studies. BJMAS. 2024;5(1):90–9. doi: 10.37745/bjmas.2022.0419

[42] Hennink MM, Kaiser BN, Weber MB. What Influences Saturation? Estimating Sample Sizes in Focus Group Research. Qual Health Res. 2019;29(10):1483–96. doi: 10.1177/1049732318821692 30628545 PMC6635912

[43] Walstra V. Picturing the group: Combining photo-elicitation and focus group methods. Anthrovision. 2020;2020(8.1).

[44] Wang C, Burris MA. Photovoice: Concept, methodology, and use for participatory needs assessment. Health education & behavior. 1997;24(3):369–87. 9158980 10.1177/109019819702400309

[45] Bill F, Olaison L. The indirect approach of semi‐focused groups: Expanding focus group research through role‐playing. Qualitative Research in Organizations and Management: An International Journal. 2009;4(1):7–26.

[46] Meherali S, Rahim KA, Campbell S, Lassi ZS. Does Digital Literacy Empower Adolescent Girls in Low- and Middle-Income Countries: A Systematic Review. Front Public Health. 2021;9:761394. doi: 10.3389/fpubh.2021.761394 34976923 PMC8716589

[47] Feroz AS. Exploring caregivers’ perspectives and perceived acceptability of a mobile-based telemonitoring program to support pregnant women at high-risk for preeclampsia in Karachi, Pakistan: A qualitative descriptive study. Healthcare. 2023. 10.3390/healthcare11030392 PMC9914365 36766967

[48] Vyas K, Louie-Poon S, Meherali S. Development of an adolescent advisory group to inform sexual and reproductive health research for first- and second-generation immigrant adolescents in Canada: A community-based participatory action research study. Front Reprod Health. 2022;4:930314. doi: 10.3389/frph.2022.930314 36388150 PMC9662937

[49] Meherali S, Louie-Poon S, Idrees S, Kauser S, Scott S, Salami B, et al. Understanding the sexual and reproductive health needs of immigrant adolescents in Canada: A qualitative study. Front Reprod Health. 2022;4:940979. doi: 10.3389/frph.2022.940979 36303669 PMC9580723

[50] Braun V, Clarke V. One size fits all? What counts as quality practice in (reflexive) thematic analysis?. Qualitative Research in Psychology. 2021;18(3):328–52.

[51] Braun V, Clarke V. Reflecting on reflexive thematic analysis. Qualitative Research in Sport, Exercise and Health. 2019;11(4):589–97. doi: 10.1080/2159676x.2019.1628806

[52] Campbell K, Orr E, Durepos P, Nguyen L, Li L, Whitmore C, et al. Reflexive Thematic Analysis for Applied Qualitative Health Research. TQR. 2021. doi: 10.46743/2160-3715/2021.5010

[53] Edwards-Jones A. Qualitative data analysis with NVIVO. Taylor & Francis. 2014.

[54] Corbin J, Strauss A. Basics of qualitative research: Techniques and procedures for developing grounded theory. Sage publications. 2014.

[55] Braun V, Clarke V. Using thematic analysis in psychology. Qualitative Research in Psychology. 2006;3(2):77–101. doi: 10.1191/1478088706qp063oa

[56] Burkett I. An introduction to co-design. Sydney: Knode. 2012.

[57] Slattery P, Saeri AK, Bragge P. Research co-design in health: a rapid overview of reviews. Health Res Policy Syst. 2020;18(1):17. doi: 10.1186/s12961-020-0528-9 32046728 PMC7014755

[58] Grindell C, Sanders T, Bec R, Mary Tod A, Wolstenholme D. Improving knowledge mobilisation in healthcare: a qualitative exploration of creative co-design methods. Evidence & Policy. 2022;18(2):265–90. doi: 10.1332/174426421x16436512504633

[59] Duea SR, Zimmerman EB, Vaughn LM, Dias S, Harris J. A Guide to Selecting Participatory Research Methods Based on Project and Partnership Goals. J Particip Res Methods. 2022;3(1):10.35844/001c.32605. doi: 10.35844/001c.32605 35799626 PMC9258244

[60] Löhr K, Weinhardt M, Sieber S. The “World Café” as a Participatory Method for Collecting Qualitative Data. International Journal of Qualitative Methods. 2020;19. doi: 10.1177/1609406920916976

