# Supplementary material for: Why are women not online? A study protocol to understand gender-based digital exclusion among marginalized women in Pakistan and promote digital gender inclusion
Source: PLoS One. 2026 Aug 20;21(8):e0356104. doi: 10.1371/journal.pone.0356104 (PMC13492796; doi:10.1371/journal.pone.0356104)
Supplement: Supplementary file 1 [file pone.0356104.s001.docx]

**S1 Appendix:** Photo-Elicitation Focus Group Discussion Guide For Marginalized Women

**Introduction:** *Asalamo-alaikum, my name is --------- from Aga Khan University, and today we will be exploring about your views on women’s access to and use of technology in their communities. This interview may take 60- 90 minutes, and instead of just asking questions, we would like to show you some pictures on those I will ask questions related to the scenario at the end. Thank you for agreeing to be part of this interview. This interview will be recorded, and my colleague will take notes to ensure that we do not miss any valuable information you share. Everything you will tell me will stay strictly confidential. Your participation is voluntary, and you can choose not to answer any specific question. There are no right or wrong answers. I am interested in your views and experiences.*

*Do you have any questions before we start?*

*Should we start the interview?*

**Section A: Socio-demographic Information**

*One form per participant*

| **Participant study ID** | ______________________________ |
| --- | --- |
| **FGD group ID / Date** | Group: ____________ Date: ____ / ____ / ________ |
| **Age (years) — eligible 18+** | ________ |
| **Marital status** | ☐ Married ☐ Unmarried ☐ Widowed ☐ Separated / Divorced ☐ Prefer not to say |
| **Household size / children** | People in household: ______ Children (if any): ______ |
| **Religion (optional)** | ☐ Muslim ☐ Christian ☐ Other ☐ Prefer not to say |
| **Years living in Azam Basti** | ________ |
| **Literacy** | ☐ Cannot read or write ☐ Can read only ☐ Can read and write |
| **Highest education completed** | ☐ None ☐ Some primary ☐ Primary ☐ Middle ☐ Secondary  ☐ Above ___________ |
| **Employment** | ☐ Home-based domestic worker ☐ Other informal work  ☐ Not in paid work ☐ Other: ____________ |
| **Main source of household income** | ☐ Self ☐ Husband / male partner ☐ Other family member ☐ Other: ______ |
| **Marginalization indicators (tick all that apply)** | ☐ Low household income (<43k) ☐ Informal employment ☐ Financially dependent on family  ☐ No personal mobile phone ☐ Shared / limited access to a phone or internet ☐ Lack of Agency in phone use ☐ Disability |
| **Mobile phone access** | ☐ Owns a personal phone ☐ Uses a shared family phone ☐ Borrows a phone ☐ No access  If owns / uses: ☐ Basic phone ☐ Smartphone |
| **Mobile internet use** | ☐ Never ☐ Rarely ☐ Sometimes ☐ Often  If often & sometimes: ☐ WIFI connection _____________  ☐ Mobile Data _______ |
| **Who usually decides about her phone / internet use** | ☐ Self ☐ Husband / male partner ☐ Father / brother ☐ Mother-in-law / elder woman  ☐ Other: ______ ☐ Prefer not to say |

**Section B: FGD with Marginalized women:**

**Image 1: Technology as a non-neutral artefact**

**Prompts:**

- What do you see here?
- Which of these do women in your home or neighbourhood use? Who uses which kind?
- Is there a difference in who is given a basic phone and who is given a smartphone? Why might that be?
- What would change if that distinction were not made?

**Image 2: Gender roles and the ordering of time**

**Prompts:**

- What is happening in this picture? Where is the phone, and where is she? Is this an ordinary moment or an unusual one?
- Does your day look like this? · When in your day could you sit down and use a phone? · Has anyone commented when you have picked up a phone during work?
- Is a woman using a phone noticed differently from a man doing the same?
- Is using a phone considered rest, or considered neglect? ·
- Does the answer change with her age, or whether she is married? What would have to shift in a day's work for it not to be noticed?

**Image 3: Mobility restrictions**

**Prompts:**

- What kind of place is this, and who is there? · Who is missing from this picture? · What would happen if a woman walked in?
- Who buys the phone, the SIM, or the credit in your household? · What happens when a phone breaks — who takes it? · Have you ever gone to such a shop yourself?
- Why don’t you see women on these shops themselves? · Is it distance, safety, reputation, or something else? · Does needing someone else to buy for you affect what you end up with? · What would make it possible for a woman to go?

**Image 4: Decision-making authority**

**Prompts:**

- Who are these people, and what is happening? · Whose phone is on the floor? · Who in this picture will speak first?
- Who in your family would decide about this phone? · Has anyone in your family asked for a phone and been refused? What reason was given? · If you wanted a phone of your own, who would you approach first?
- Why do those particular people decide? ·
- Are there women whose decisions about technology are respected here? What gives them that standing? ·
- Do older women and men decide about different things? · Whose view would have to change first for the decision to be different?

**Image5: Surveillance and control**

**Prompts:**

- What is happening between these two people? · Is one showing, or one checking? How can you tell? · Does this look ordinary to you?
- Do people in families here look at one another's phones? · Is there a password on any phone you use?
- Has something on a phone ever been seen by someone it was not meant for?
- Is checking normal in some households and not in others?
- What does it mean here if a woman has a password? ·
- Does knowing you might be checked change what you do on a phone?
- What would need to change for checking to stop?

**Image 6: Mutual shaping and change over time**

**Prompts:**

- What is this woman doing? · How does she seem — comfortable, hurried, hidden? · Is anyone else present?
- Have you ever had a moment like this? · Has anything about women and phones here changed in the last five years?
- Why is it possible for her and not for others? ·
- What made it possible — money, family, work, age, something else? ·
- Has a phone changed anything in a household here, in how money or children or contact are handled?
- What could help in making it happen more widely?
