# Supplementary material for: Why are women not online? A study protocol to understand gender-based digital exclusion among marginalized women in Pakistan and promote digital gender inclusion
Source: PLoS One. 2026 Aug 20;21(8):e0356104. doi: 10.1371/journal.pone.0356104 (PMC13492796; doi:10.1371/journal.pone.0356104)
Supplement: Supplementary file 2 [file pone.0356104.s002.docx]

**S2 Appendix:** Role-Play Focus Group Discussion Scenarios For Family Gatekeepers and Community Leaders

**Introduction:** *Asalamo-alaikum, my name is --------- from Aga Khan University, and today we will be exploring about your views on women’s access to and use of technology in their communities. This interview may take 90-120 minutes, and instead of just asking questions, I would like you to act out a situation I will ask questions related to the scenario at the end. Thank you for agreeing to be part of this interview. This interview will be recorded, and my colleague will take notes to ensure that we do not miss any valuable information you share. Everything you will tell me will stay strictly confidential. Your participation is voluntary, and you can choose not to answer any specific question. There are no right or wrong answers. I am interested in your views and experiences.*

*Do you have any questions before we start?*

*Should we start the interview?*

**Section A: Socio-demographic Information:**

*One form per participant*

| **Participant study ID** | ______________________________ |
| --- | --- |
| **FGD group ID / Date** | Group: ____________  Date: ____ / ____ / ________ |
| **Participant category** | ☐ Family gatekeeper ☐ Community leader |
| **If family gatekeeper** | ☐ Male partner ☐ Brother ☐ Other male relative ☐ Elderly woman |
| **If community leader** | ☐ Local organization member ☐ Community elder  ☐ Local influencer ☐ Other: ____________ |
| **Age (years)** | _____________ |
| **Gender** | ☐ Male ☐ Female ☐ Prefer not to say |
| **Marital status** | ☐ Married ☐ Unmarried ☐ Widowed ☐ Other / Prefer not to say |
| **Religion (optional)** | ☐ Muslim ☐ Christian ☐ Other _________ |
| **Years living / working in Azam Basti** | ________ |
| **Highest education completed** | ☐ None ☐ Primary ☐ Middle ☐ Secondary ☐ Higher sec. ☐ Above _______ |
| **Main occupation** |  |
| **Mobile phone ownership** | ☐ Yes, personal ☐ Shared ☐ No  If yes: ☐ Basic phone ☐ Smartphone |
| **Uses mobile internet** | ☐ Yes, regularly ☐ Sometimes ☐ No  If yes: ☐ WIFI connection _____________ ☐ Mobile Data _______ |
| **Gatekeepers: relationship to a woman whose technology access you influence** | ________________________  (e.g., wife, sister, daughter, daughter-in-law) |
| **Community leaders: nature of role / how recognised** |  |
| **Involvement in decisions about women’s technology use** | ☐ Often ☐ Sometimes ☐ Rarely ☐ Never |

**Section B: Role-play Scenarios**

**1. “A phone in the house”**

### **Facilitator introduction**

*“We are going to try something a little different. Instead of me asking questions, I would like you to act out a situation that families deal with. Each of you will take a role it does not have to be your real life. Just imagine you are this family, sitting together, and this matter has come up. Speak and respond as that person might. There are no wrong answers, and you do not have to reach an agreement.”*

### **The situation:**

A woman in the household has a concrete, everyday reason to use a mobile phone. The family is sitting together one evening and the matter comes up: should she have access to a phone, whose phone, how, and on what terms? The family talks it over.

Trigger: The health center has asked her to receive appointment and medicine reminders by SMS

**Roles (skeletal cards) can be more than 4**

*Each card offers only a sketch. Participants fill in the character from their own experience.*

| **Role** | **Sketch (skeletal — participant fills it in from lived experience)** |
| --- | --- |
| **Husband / male partner** | Responsible for the household; weighing cost, safety, what is considered appropriate, and what others may say. |
| **Brother** | Close to the woman; concerned with the family’s reputation and how the community might perceive the decision. |
| **Older male relative (e.g., father-in-law or uncle)** | A senior authority in the household whose view carries weight; tends toward established practice. |
| **Elderly woman (e.g., mother-in-law)** | Influential in household matters and in shaping younger women’s access; may hold protective or cautious views. |

### **The task (read aloud)**

*“Please talk through this situation as the family would. There is no right answer, and you do not need to agree. Take your time.”*

### **Enactment guidance**

- Allow 15–20 minutes. The facilitator stays out of the scene.
- If the scene stalls, a light prompt only, e.g., “And what does [role] think about that?
- The note-taker records the dynamics: who defers to whom, what reasons are offered, and what is treated as settled versus open.

### **Debrief prompts**

- How did it feel to play your role?
- How close was that conversation to ones that really happen in families here?
- Which reasons carried the most weight in the discussion? Why those?
- Who had the most say in the end, and how did that happen?
- What would make it easier, or safer, for a woman in this situation to use a phone?
- What would need to change for the decision to be made differently?
- In a situation like this, how might the woman’s own view be heard?
- Are there ways a family could support a woman’s phone use that we did not see in the scene?

**2. “A skills session for women”**

### **Facilitator introduction**

*“We are going to act out a meeting. Imagine you are a group of respected individuals in the community, and this proposal has been presented to you for your consideration. Each of you will take a role, it does not have to reflect your own opinion. Speak as that person might. There are no wrong answers, and you do not need to reach a decision.”*

### **The situation (read aloud or handed out)**

A local organisation proposes to hold a short, women-only mobile-phone and digital-skills session for interested women at a community venue. The proposal is brought to a gathering of community leaders and respected gatekeepers for their views before anything is decided. The group talks it over: would they support it, under what conditions, and what concerns would they want addressed?

### **Roles (skeletal cards)**

*Each card offers only a sketch. Participants fill in the character from their own experience.*

| **Role** | **Sketch (skeletal — participant fills it in from lived experience)** |
| --- | --- |
| **Respected community elder / leader** | Concerned with the community’s standing and harmony; weighs potential benefits against possible objections. |
| **Local organization member** | Brings the proposal; sees potential benefits such as access to information and services; wants community buy-in. |
| **Gatekeeper voicing common concerns** | Raises questions of safety, reputation, and appropriateness on behalf of families. |
| **Community influencer / moral authority** | Concerned with cultural and religious appropriateness; influential in shaping community opinion. |

### **The task (read aloud)**

*“Please discuss this proposal as this group of community figures would. You do not need to agree or reach a decision the aim is to talk it through.”*

### **Enactment guidance**

- Allow 15–20 minutes; the facilitator intervenes minimally.
- The note-taker records which conditions and concerns are raised, whose voice carries weight, and how women’s interests are represented — or not — in the discussion.

### **Debrief prompts**

- How did it feel to take these roles?
- How similar was that to how such matters are actually discussed in the community?
- What concerns came up most strongly? What sat behind them?
- What conditions would make something like this acceptable here?
- What part could community leaders play in how women’s technology use is viewed?
- How could women themselves take part in a decision like this?
- What might change the way the community thinks about women using mobile phones?

**3. “An Emergency at Home”**

### **Facilitator introduction**

*“We are going to act out a situation that families sometimes face. Imagine you are this family, sitting together after a difficult day, talking over what happened and what should be done. Each of you will take a role — it does not have to be your real life. Speak as that person might. There are no wrong answers, and you do not have to reach agreement.”*

### **The situation (read aloud or handed out)**

A woman is at home caring for a sick child while other family members are away. The child suddenly becomes very ill and requires immediate medical attention. She needs to contact a family member, health worker, or transportation service but does not own a mobile phone and cannot easily access one.

Some time later, the family and community members discuss what should happen and whether women should have direct access to a phone during emergencies.

### **Roles (skeletal cards)**

*Each card offers only a sketch. Participants fill in the character from their own experience.*

| Role | Sketch (skeletal — participant fills it in from lived experience) |
| --- | --- |
| Husband/male partner | Was away when the child fell ill; weighs cost, safety, control, and what happened. |
| Brother | Concerned about family reputation and protecting family members. |
| Elderly woman | Experienced caregiver who may have traditional or protective views. Senior in the household and influential over how matters are handled; may hold protective or cautious views. |
| Community leader | Thinks about wider community support and available resources. Someone the family turns to; weighs the community’s standing and what is considered acceptable. |
| Fifth role (optional): | Neighbor: Familiar with local support systems and community responses. the CAC may add a female relative (e.g., a sister-in-law) |

### **The task (read aloud)**

“Please talk through this situation *what happened and what should happen* as the family and community members would. There is no right answer, and you do not need to agree. Take your time.”

### **Enactment guidance**

- Allow 15–20 minutes.
- The facilitator intervenes minimally.
- If discussion stalls, use light prompts such as: “What would [role] suggest in this situation?”
- The note-taker records decision-making patterns and whose voices carry the most weight.

### **Debrief prompts**

- How did it feel to take your role?
- How close was this to situations families here have faced?
- In the discussion, what mattered most when weighing whether a woman should have a phone in an emergency?
- What are the risks when a woman at home cannot reach anyone? Who carries those risks?
- What arrangements could make sure help can be reached quickly — for the woman, the children, and the whole family?
- What would need to change for a woman to use a phone freely when it is urgent?
- Are there ways the family could support this that we did not see in the scene?
