# Supplementary material for: Why are women not online? A study protocol to understand gender-based digital exclusion among marginalized women in Pakistan and promote digital gender inclusion
Source: PLoS One. 2026 Aug 20;21(8):e0356104. doi: 10.1371/journal.pone.0356104 (PMC13492796; doi:10.1371/journal.pone.0356104)
Supplement: Supplementary file 3 [file pone.0356104.s003.docx]

**S3 Appendix:** In-Depth Interview Guide For Marginalized Women

**Introduction:** *Asalamo-alaikum, my name is --------- from Aga Khan University, and today we will be exploring about your views on women’s access to and use of technology in their communities. This interview may take 40-60minutes. Thank you for agreeing to be part of this interview. This interview will be recorded, and my colleague will take notes to ensure that we do not miss any valuable information you share. Everything you will tell me will stay strictly confidential. Your participation is voluntary, and you can choose not to answer any specific question. There are no right or wrong answers. I am interested in your views and experiences.*

*Do you have any questions before we start?*

*Should we start the interview?*

**Section A:  Socio-demographic Information**

*One form per participant*

| **Participant study ID /** |  | ______________________________ |
| --- | --- | --- |
| **FGD group ID / Date** |  | Group: ____________     Date: ____ / ____ / ________ |
| **Age (years) — eligible 18+** |  | ________ |
| **Marital status** |  | ☐ Married   ☐ Unmarried   ☐ Widowed   ☐ Separated / Divorced   ☐ Prefer not to say |
| **Household size / children** |  | People in household: ______        Children (if any): ______ |
| **Religion (optional)** |  | ☐ Muslim   ☐ Christian   ☐ Other   ☐ Prefer not to say |
| **Years living in Azam Basti** |  | ________ |
| **Literacy** |  | ☐ Cannot read or write   ☐ Can read only   ☐ Can read and write |
| **Highest education completed** |  | ☐ None   ☐ Some primary   ☐ Primary   ☐ Middle   ☐ Secondary   ☐ Above ___________ |
| **Employment** |  | ☐ Home-based domestic worker   ☐ Other informal work  ☐ Not in paid work   ☐ Other: ____________ |
| **Main source of household income** |  | ☐ Self   ☐ Husband / male partner   ☐ Other family member   ☐ Other: ______ |
| **Marginalization indicators (tick all that apply)** |  | ☐ Low household income (<43k)  ☐ Informal employment   ☐ Financially dependent on family  ☐ No personal mobile phone   ☐ Shared / limited access to a phone or internet ☐ Lack of Agency in phone use ☐ Disability |
| **Mobile phone access** |  | ☐ Owns a personal phone   ☐ Uses a shared family phone   ☐ Borrows a phone   ☐ No access  If owns / uses:  ☐ Basic phone   ☐ Smartphone |
| **Mobile internet use** |  | ☐ Never   ☐ Rarely   ☐ Sometimes   ☐ Often  If often & sometimes:  ☐  WIFI connection _____________   ☐  Mobile Data _______ |
| **Who usually decides about her phone / internet use** |  | ☐ Self   ☐ Husband / male partner   ☐ Father / brother   ☐ Mother-in-law / elder woman  ☐ Other: ______   ☐ Prefer not to say |

**SECTION B: Interview Questions**

- ***Everyday Life***

1. **To begin, could you tell me a little about a typical day for you?**

- What are the main things that fill your day?
- Who else lives in your household?

2. **Where does technology, such as a mobile phone, fit into your daily life, if at all?**

- Whose phone is it, and how did it come to be used that way?
- At what time of day, if any, would you be able to sit with it?
- ***Access to and Use of Technology***

3. **Can you describe your own experience of using a mobile phone or the internet?**

- What do you use it for, and how often?
- What kind of phone is it — a basic handset or a smartphone? Who has which, in your household?
- If you do not use one, can you tell me about that?

4. **How did you first come to use (or not use) a phone?**

- What made it possible, or difficult?
- Has this changed over time in any way?

5. **Since phones came into your household, has anything else changed?**

- Has it changed how money is handled, how children are managed, or who talks to whom?
- Has having, or not having, a phone changed how you are seen by others in your family?

6. **Who pays for the phone in your household, and who pays for credit or data?**

- Does the person who pays decide how it is used?
- When money is short in the household, what gets cut first? Where does credit or data come in that order?

7. **If a phone or credit cost less, would that change things for women here — or is cost not the main thing?**

- Do you know families who could afford a phone for a woman but do not provide one? What is the reason there?
- ***Gender Roles***

8. **In your community, is there any difference in how a woman using a phone is seen compared with a man using one?**

- Can you describe that difference?
- Where do you think those expectations come from?

9. **Are there things on a phone that are considered acceptable for women here, and things that are not?**

- Calls, messages, voice notes, internet, social media — are these seen differently from one another?
- Is the concern about the phone itself, or about what can be done on it?

10. **Does having or using a phone affect how a young woman’s family is spoken about?**

- Does it affect marriage prospects, in either direction?
- Is the concern about what she does, or about what people will say?
- ***Mobility***

11. **How do women you know obtain a phone, mobile credit or data, or repairs?**

- Are women able to go to shops for these things themselves?
- If not, who does this on their behalf, and how is it arranged?
- Does depending on someone else to buy affect what you end up with?
- What would happen if a woman went to such a shop herself?
- Is the difficulty distance, safety, reputation, or something else?

12. **Do restrictions on where women can go affect their use of technology in any way?**

- Can you give me an example from your own experience?
- Does having a phone make it easier or harder to move around? Does it change who needs to know where you are?
- ***Decision-Making Authority***

13. **In families in this community, who usually decides whether a woman can use a phone?**

- How is that decision usually made?
- What reasons are given?
- Do women in this community have a say in whether other women can use a phone?
- Do older women and men have a say over different things?

14. **In your household, who decides whether a woman can use a phone?**

- Is it discussed, or simply understood?
- Has anyone in your family asked for a phone and been refused? What reason was given?
- If you wanted a phone of your own, who would you approach first, and what would you say?
- Has a decision like this ever changed — for you or for someone you know?
- What made it change?
- ***Surveillance or Control of Technology Use***

15. **Do people in families here look at one another’s phones?**

- Is this expected, or taken as a sign that something is wrong?
- Does it happen between spouses, between parents and children, or both?

16. **Once a woman has access to a phone, is the way she uses it watched or guided by anyone?**

- How does that happen, and by whom?
- How do women feel about this, in your experience?
- Are there passwords on phones here? What does it mean if a woman has one?

17. **Are there times, places, or ways in which a woman’s phone use is limited?**

- What lies behind those limits, as you understand it?

18. **Do women here worry about anything happening to them through a phone? What sorts of things?**

- Unwanted calls or messages, photographs, contact from strangers — do these come up?
- Have you heard of a woman in this community having trouble because of a phone?
- What happened afterwards — for her, and for her access to the phone?
- ***Mutual shaping and change over time***

19. **Is there anything you wanted to do on a phone but did not know how to do?**

- How did you learn what you do know? Who taught you?
- Who would you ask if you were stuck?
- Do you need to read and write to use a phone the way you would want to? Does that affect what you use it for?
- Do you use voice messages or calls rather than typing? Why?
- Are there women in this community who help others learn to use phones?

20. **Since phones came into your household, has anything else changed?**

- Has it changed how money is handled, how children are managed, or who talks to whom?
- Has having, or not having, a phone changed how you are seen in your family?

21. **If you could use a phone freely, what would you use it for?**

- Is there anything you have needed — health information, contacting family, money, work — that a phone would have helped with?
- Has not having one ever cost you something?

22. **What would need to be different in this community for women to use phones more freely?**

Who would need to think differently, and what would persuade them? What would change first?

SECTION 8. Closing

23. Looking back over everything we have discussed, is there anything you would like to add?

Is there anything important about women and technology in your community that I did not ask about but that you feel I should know?
