# Supplementary material for: Why are women not online? A study protocol to understand gender-based digital exclusion among marginalized women in Pakistan and promote digital gender inclusion
Source: PLoS One. 2026 Aug 20;21(8):e0356104. doi: 10.1371/journal.pone.0356104 (PMC13492796; doi:10.1371/journal.pone.0356104)
Supplement: Supplementary file 4 [file pone.0356104.s004.docx]

**S4 Appendix:** In-Depth Interview Guide For Family gatekeepers and Community leaders

**Introduction:** *Asalamo-alaikum, my name is --------- from Aga Khan University, and today we will be exploring about your views on women’s access to and use of technology in their communities. This interview may take 40-60minutes. Thank you for agreeing to be part of this interview. This interview will be recorded, and my colleague will take notes to ensure that we do not miss any valuable information you share. Everything you will tell me will stay strictly confidential. Your participation is voluntary, and you can choose not to answer any specific question. There are no right or wrong answers. I am interested in your views and experiences.*

*Do you have any questions before we start?*

*Should we start the interview?*

**Section A: Socio-demographic Information:**

*One form per participant*

| **Participant study ID /** | ______________________________ |
| --- | --- |
| **FGD group ID / Date** | Group: ____________     Date: ____ / ____ / ________ |
| **Participant category** | ☐  Family gatekeeper      ☐  Community leader |
| **If family gatekeeper** | ☐  Male partner   ☐  Brother   ☐  Other male relative   ☐  Elderly woman |
| **If community leader** | ☐  Local organization member   ☐  Community elder  ☐  Local influencer   ☐  Other: ____________ |
| **Age (years)** | _____________ |
| **Gender** | ☐  Male    ☐  Female    ☐ Prefer not to say |
| **Marital status** | ☐  Married   ☐  Unmarried   ☐  Widowed   ☐  Other / Prefer not to say |
| **Religion (optional)** | ☐  Muslim   ☐  Christian   ☐ Other   _________ |
| **Years living / working in Azam Basti** | ________ |
| **Highest education completed** | ☐ None   ☐ Primary   ☐ Middle   ☐ Secondary   ☐ Higher sec.   ☐ Above _______ |
| **Main occupation** |  |
| **Mobile phone ownership** | ☐  Yes, personal   ☐  Shared   ☐  No  If yes:  ☐  Basic phone   ☐  Smartphone |
| **Uses mobile internet** | ☐  Yes, regularly   ☐  Sometimes   ☐   No  If yes:  ☐  WIFI connection _____________   ☐  Mobile Data _______ |
| **Gatekeepers: relationship to a woman whose technology access you influence** | ________________________   (e.g., wife, sister, daughter, daughter-in-law) |
| **Community leaders: nature of role / how recognised** |  |
| **Involvement in decisions about women’s technology use** | ☐ Often   ☐ Sometimes   ☐ Rarely   ☐ Never |

**SECTION B: Interview Questions**

- ***Role in Family and Community***

1. **To begin, could you tell me a little about your role in your family and community?**

- Who do you live with, and what responsibilities do you hold?
- For community leaders: what kind of matters do people bring to you?

2. **How common are mobile phones and the internet among households you know?**

- Who usually owns a phone? Who usually has to borrow one?
- What kinds of phones are commonly used (smartphone/basic phone)?
- Who usually pays for phone credit or internet packages?
- Who tends to use them, and for what?
- ***Perceptions of Women’s Access to and Use of Technology***

3. **In your view, how do women in your community access and use technology such as mobile phones?**

- What do you think are the benefits, if any?
- What concerns, if any, do you or others have?
- How do women usually learn to use mobile phones or the internet?
- Who usually teaches or supports them?
- Are there women who would like to learn but do not have the opportunity? Why?

4. **Besides family attitudes, are there other things that make it difficult for women to access or use mobile phones or the internet?
   -** affordability, phone ownership, internet costs, electricity, network coverage, literacy.
5. **Some families here allow women to use phones freely and others do not. What accounts for the difference?**

- Is it money, education, work, who is in the household, something else?
- Are there families where it was once not allowed and now is? What changed, and who changed their mind first?
- Are there reasons a family might decide a woman should have a phone — work, children's schooling, a relative abroad, health, emergencies?
- Which of those reasons carries the most weight with families here?
- Can you think of a woman whose use of a mobile phone has benefited her or her family? Could you tell me about that?

6. **How is a woman using a phone generally viewed among the people you know?**

- Does this view differ from how a man using a phone is seen? In what way?
- Does the reaction differ depending on the woman's age, marital status, education, or employment?
- Are there particular activities that are considered acceptable or unacceptable for women to do on a phone?
- ***Gender Roles and Cultural Narratives***

7. **What shapes the way people here think about women using technology?**

- What part do family customs, values, or beliefs play?
- Do you think these expectations are changing at all? How?

8. **Do you think use of mobile phones has changed women's roles, relationships, or responsibilities within families or the community? In what ways?**

- ***Decision-Making Authority***

9. **Consider a situation where a decision is being made about whether a woman in a family can use a phone. In your experience, how does a decision like that usually come about?**

- Who is typically involved?
- What reasons carry the most weight?
- Does the decision differ depending on who bought or pays for the phone?

10. **What influences the way a man in that position thinks about the decision?**

- Can you describe what he might be weighing?

11. **In families you know, what part do women play in these decisions — mothers, mothers-in-law, elder sisters?**

- Whose view carries weight, and what gives her that standing?
- Do older women and older men have a say over different things?
- Have you seen a woman in a family argue for, or against, another woman having a phone?
- Does it differ for a daughter compared with a daughter-in-law?

12. **And in your own household, how does this work?**

- Who has a phone, and how did that come about?
- Has a request ever been made that you did not agree to? What was the reason?
- Has your own thinking on this changed over the years?
- ***Safety, Reputation, and Control of Use***

13. **Concerns about safety or reputation are sometimes raised in relation to women’s phone use. In your experience, what lies behind such concerns?**

- Can you give me an example of a concern you have heard?
- Are there situations where using a phone is encouraged rather than discouraged?

14. **If something does go wrong — an unwanted contact, a photograph, a rumour — what happens afterwards?**

- What happens for the woman, and what happens for her family?
- Does it affect how the family is spoken about, or a young woman's marriage prospects?
- Does she keep the phone afterwards?
- Is the concern more about what actually happens, or about what people will say?

15. **How are these concerns usually managed within families?**

- What does that mean for how a woman is able to use a phone day to day?
- Who tends to oversee or guide her use?

16. **Do people in families here look at one another's phones?**

- Is that expected, or is it taken as a sign that something is wrong?
- Does it happen between spouses, between parents and children, or both?
- Are there passwords on phones here? What does it mean if a woman has one?
- What happens if something is found that was not expected?
- ***Mobility***

17. **How do women's daily routines, responsibilities, or ability to travel affect their opportunities to learn about or use technology??**

- What part do family members play in this?
- ***Reflection on Change***

18. **Has women's use of phones ever come up as a matter for the community rather than for individual families?**

- Has it been raised in any gathering, committee, or local organisation? What was said?
- Are there any shared understandings or informal rules here about it?
- Do people give religious reasons when they discuss women and phones? What is said?
- Do different families in this community see it differently?

19. **What, if anything, could change decisions or attitudes about women’s use of technology in your community?**

- What might a male family member, such as a brother or partner, do to support a woman in using mobile technology?
- What shifts in thinking would need to happen for this to be more widely accepted?
- What role, if any, can community leaders, health workers, schools, or local organisations play in supporting women's safe and meaningful use of technology?
- ***Closing***

20. **Looking back over everything we have discussed, is there anything you would like to add? Is there anything important about women and technology in your community that I did not ask about but that you feel I should know?**

*Thank the participant, explain the next steps in the study (including the Phase 3 co-design workshops)*
