# Supplementary material for: Why are women not online? A study protocol to understand gender-based digital exclusion among marginalized women in Pakistan and promote digital gender inclusion
Source: PLoS One. 2026 Aug 20;21(8):e0356104. doi: 10.1371/journal.pone.0356104 (PMC13492796; doi:10.1371/journal.pone.0356104)
Supplement: Supplementary file 5 [file pone.0356104.s005.docx]

**S5 Appendix:** Co-design Workshop Guide

**Purpose:**
To collaboratively develop and refine strategies to promote women’s access to and meaningful use of mobile technology by addressing sociocultural barriers identified in Phase 2.

**Activity 1: Reflection on Phase 2 Findings**

**Purpose:** Identify and validate key barriers and facilitators influencing women’s digital access.

**Discussion prompts:**

- What barriers make it difficult for women in our community to access or use mobile technology?
- Which of these barriers are most important to address?
- What changes are needed at the family or community level to support women’s digital inclusion?

**Activity 2: Brainstorming Session – Developing Strategies**

**Purpose:** Generate potential strategies to address identified barriers.

**Discussion prompts:**

- What can families, communities, or organizations do to support women’s access to mobile technology?
- What strategies could encourage safe and meaningful use of mobile phones among women?
- How can men and other family members support women’s use of digital technologies?
- What community-level changes could make digital access more acceptable for women?

**Activity 3: World Café Dialogue – Refining Strategies**

**Purpose:** Further develop and refine proposed strategies through collective discussion.

**Discussion prompts:**

- What are the strengths and potential benefits of this strategy?
- What challenges or concerns might arise in implementing this strategy?
- How can this strategy be adapted to fit our community context?
- Who should be involved in supporting this change?
- What would make this strategy acceptable and sustainable?

**Closing Reflection**

- Which strategies do you think have the greatest potential to improve women’s digital access?
- What changes would you like to see in your community regarding women’s use of technology?
